# Consumer Acceptance of Brown and White Rice Varieties

**DOI:** 10.3390/foods10081950

**Published:** 2021-08-22

**Authors:** Tanweer Aslam Gondal, Russell S. J. Keast, Robert A. Shellie, Snehal R. Jadhav, Shirani Gamlath, Mohammadreza Mohebbi, Djin Gie Liem

**Affiliations:** 1 CASS Food Research Centre, School of Exercise and Nutrition Sciences, Deakin University, Burwood Campus 221 Burwood Highway, Burwood, VIC 3125, Australia; tgondal@deakin.edu.au (T.A.G.); russell.keast@deakin.edu.au (R.S.J.K.); robert.shellie@deakin.edu.au (R.A.S.); Snehal.Jadhav@deakin.edu.au (S.R.J.); shirani.gamlath@deakin.edu.au (S.G.); 2 Institute of Food Science and Nutrition, Bahauddin Zakariya University, Multan 66000, Pakistan; 3 Biostatistics Unit, Faculty of Health, Deakin University, Geelong, VIC 3125, Australia; m.mohebbi@deakin.edu.au

**Keywords:** brown rice, white rice, sensory, consumer acceptance, Just About Right scale, JAR, penalty analysis

## Abstract

Rice is consumed as a staple food by more than half of the world’s population. Due to a higher fibre and micronutrient content, brown rice is more nutritious than white rice, but the consumption of brown rice is significantly lower than that of white rice, primarily due to sensory attributes. Therefore, the present research aimed to identify the sensory attributes which drive liking of Australian-grown brown and white rice varieties. Participants (*n* = 139) tasted and scored (9-point hedonic scale) their liking (i.e., overall liking, aroma, colour and texture) of brown and white rice types of Jasmine (Kyeema), Low GI (Doongara), and Medium grain rice (Amaroo). In addition, participants scored aroma, colour, hardness, fluffiness, stickiness, and chewiness, on Just About Right Scales. A within-subjects crossover design with randomised order (William’s Latin Square design) was used with six repeated samples for liking and Just About Right scales. Penalty analyses were applied to determine the relative influence of perception of sensory attributes on consumer liking of the rice varieties. Across all varieties, white rice was liked more than brown rice due to the texture and colour, and Jasmine rice was preferred over Low GI and Medium Grain. Rice texture (hardness and chewiness) was the most important sensory attribute among all rice varieties and aroma was important for driving of liking between white rice varieties.

## 1. Introduction

Rice is consumed as a staple food by more than 4 billion people around the globe [1,2,3]. Rice is a significant source of dietary nutrients such as carbohydrates, vitamins, and minerals [4,5]. For populations that rely on rice as a staple food, it delivers approximately 21% of the consumed energy and 15% of the consumed protein [6].

Australia produces high quality rice from different varieties, which are categorised as aromatic Thai jasmine origin and non-aromatic rice [7]. Aromatic rice varieties have distinctive popcorn like flavour notes due to the presence of 2-acetyl-1-pyrroline [7,8,9,10]. Furthermore, rice can be classified based on the milling process. The milling of the whole grain results in brown rice, and a further removal of bran and germ results in white rice. [11]. Although white rice is more commonly consumed, brown rice is considered healthier due to nutritional components such as lipids, proteins, dietary fibre, and polyphenols [12,13].

The sensory profile of rice is an important driver of consumer acceptance. Sensory attributes have a strong influence on product selection, consumption, and purchase decisions [14,15]. Sensory attributes such as physical appearance (i.e., uniformity, cleanliness, brightness, glossiness and translucency of the rice grain) [16], taste (e.g., sweetness, bitterness), and aroma (e.g., floral notes) are drivers of liking [17] that affect consumer acceptance of rice.

Furthermore, rice texture (i.e., cohesiveness, softness) has been suggested to be of high importance for consumer acceptance of rice. A previous study reported that brown rice texture was less liked compared to white rice and there was variation in liking of the various textures of different brown rice varieties [18]. Along the same lines, Suwansri et al. suggested that an increase in the hardness of rice is associated with a lower consumer acceptability [19]. The importance of texture has also been emphasised by Maleki et al., who suggests that consumers can be segmented based on their preference for different rice textures [20]. In their study, fluffiness was a driver of liking for the majority of consumers (44%), whereas for smaller segments of consumers, liking was mainly driven by flavour attributes.

Within each rice variety, the milling process (e.g., white vs. brown rice) alters the nutrient composition and sensory attributes [21]. For example, brown rice has a higher lipid content compared with white rice. The lipid context affects the sensory profile due to lipid oxidation in the bran layer of brown rice [22]. Lipid oxidation leads to the development of off flavours [23], which potentially impact consumer perception and acceptance. In short, differences in the acceptance of white and brown rice are likely caused by differences in sensory profiles, which are related to differences in nutrient composition [24].

In Australia, 90% of rice is consumed as white rice, whereas only 10% is consumed as brown rice [25], which is similar to global rice consumption patterns [25,26]. Brown rice is considered a healthier option than white rice [27]. To understand what drives the difference in consumption of brown and white rice, it is important to investigate the sensory differences of brown and white rice.

The objective of this study was to identify the drivers of liking of Australian grown brown and white rice varieties. It will provide important information for rice industry and breeding programmes for the development of new rice varieties to meet consumer needs.

## 2. Participants, Materials, and Methods

### 2.1. Study Design

A within-subjects crossover design with randomised order (William’s Latin Square design) for liking and Just About Right scales with six repeated samples was used in the present study. To determine the required participant sample size, G*power [Version 3.1.9.2, Franz Faul, Universitat Kiel, Kiel, Germany] was used. Based on six measurements (six rice samples) comparisons within subjects with alpha level 0.05, power of 0.8, and a small effect size (f = 0.10), the minimum sample size was 109. To account for potential dropouts, 140 participants from Consumer Analytical Safety Sensory (CASS) Food Research Centre database were recruited. Participants were excluded if they had food allergies, dietary restrictions, and/or were pregnant or lactating. Participants were asked to refrain from eating, drinking, or brushing their teeth one hour prior to testing. The rice consumer study was approved by the research ethics committee Deakin University (HEAG-H 29_2018).

### 2.2. Measurements

Participants were asked to complete two questionnaires concerning (1) demographics (age, gender, education, and marital status), and (2) rice consumption (type of rice (brown or white), number of times they eat rice daily, weekly or fortnightly, and awareness of brown rice health benefits). To assess the liking and sensory perception of the rice samples before and after tasting the rice samples, participants filled out 9-point hedonic scales (1 = extremely dislike and 9 = extremely like) [28] for overall liking, aroma, colour, and texture. In addition, participants completed Just About Right scales for aroma intensity, colour, hardness, fluffiness, stickiness, and chewiness, similar to previous published research [29]. A Just About Right scale, is a bipolar labelled attribute scale [30], which has an anchored mid-point that corresponded to Just About Right for each attribute [31]. The Just About Right scales provided the participants with 3 answer options per sensory attribute (1 = not enough, 2 = Just about Right, 3 = too much) [32].

### 2.3. Materials

Three most commonly consumed Australian rice varieties (Jasmine rice (Kyeema), Low GI (Doongara) and Medium grain (Amaroo) (Table 1)) with both brown and white rice types were sourced from Sunrice (Ricegrowers Ltd., Leeton, Australia) Australia [33].

Rice samples were washed 2 to 3 times in cold running water until the water ran clear. Rice samples were cooked in dedicated rice cookers (“Grain Master” HD4514/72_ UM_ US_v1.0, Philips, China), to avoid cross flavour contamination, according to manufacturer’s instructions with specific water to rice ratios (Table 1). Rice samples and water quantities were measured by a measuring cup. Rice was cooked at quick rice cooking mode and kept warm at 600 C (as measured by an infrared thermometer Xintest HT-88A; Dongguan Xintai Instrument Co., Guangdong, China) in the rice cooker for no longer than the duration of the sensory test (approximately 45 min).

### 2.4. Testing Procedure

Sensory testing took place in a sensory laboratory, which consisted of partitioned booths and a high capacity air filtration system, of the CASS Food Research Centre, Deakin University, Melbourne, Australia. On arrival, participants were instructed to carefully read the Plain Language Statement and sign the consent form. Ten participants participated in each one hour session. Rice samples were served to the participants in 30 mL clear plastic medicine cups that were labelled with three digit unique codes. Each cup contained 10 g of rice and participants were instructed to consume at least one teaspoon of rice. The rice samples were randomly presented one at a time directly from the rice cooker at a temperature of 55 ± 3 °C. The participants were instructed to rinse their mouth with filtered water for five seconds and use crackers between tasting the different rice samples.

The test consisted of two parts (i.e., before tasting, after tasting). In the first part, the participants received the following instruction: “do not eat the rice samples, only look, feel (e.g., hold the rice between your fingers) and smell the rice”. Next, participants were asked to rate overall liking and their liking for aroma and colour on a 9-point hedonic scale, and fluffiness, stickiness, hardness, and aroma intensity on Just About Right Scales.

In the second part, the participants were instructed to taste the rice samples (one by one) and rate on 9-point hedonic scales, their overall liking, and texture for each rice sample. In addition, participants rated their perceived intensity of flavour, fluffiness, hardness and chewiness on Just About Right scales. There was a one minute break after the tasting of each sample to avoid tasting fatigue of the participants.

The data were collected on computers using Compusense Software Academic Consortium (Compusense, Inc., Guelph, ON, Canada). Gift vouchers (50AUD) were served to each participant on completion of the rice consumer test.

### 2.5. Statistical Analysis

All rice consumer study data were exported from Compusense Cloud into Microsoft Excel version 1708 (Microsoft Corporation) for data cleaning. For the statistical analysis of liking, the program Stata/IC 15.0 (StataCorp LLC, 4905 Lakeway Drive, College Station, TX 77845, USA) was used. Descriptive statistics (mean, standard deviation and correlation coefficient) were calculated for overall liking scores and all sensory attributes. Box plots and scatter plots were extracted for overall liking and for other sensory attributes. Linear mixed model approach was used to analyse repeated measure Analysis of Variance (ANOVA) data to determine the effect of rice varieties (Jasmine, Low GI and medium grain rice samples) and rice types (brown, white) on overall liking, aroma, colour, and texture linking. This approach accounts for within subject autocorrelation via a random intercept in the model. The combined effect of rice varieties and types of rice was tested through a model that contained the main effects of rice type (brown and white) and varieties (Jasmine, Low GI and Medium Grain) as well as the two-way interaction between varieties, and types of rice. The post-hoc pairwise comparison (Bonferroni adjusted) was conducted to identify the significant difference in sensory attributes among rice varieties and rice types.

The descriptive statistics for Just About Right attributes, overall liking, and penalty analysis (*p* < 0.05) of brown and white rice from the three varieties were conducted in XLSTAT Sensory version 2020.3 (Addinsoft, New York, NY, USA). The penalty was a weighted difference between means (mean liking of JAR category minus the mean of liking for other two levels (too low and too high) taken together) [32]. Spearman’s correlation coefficients were calculated. Mean drop plots were extracted to identify the effect of JAR attributes on overall liking of rice. The mean drops were plotted against the percentage of consumers. For penalty analysis and mean drop plots, 20% consumers were considered as the threshold level for each JAR attribute [30].

## 3. Results

### 3.1. Participants

The participants (*n* = 140, female 52%, male 48%) from different age groups participated in the consumer study, one participant was excluded during data cleaning because of incomplete rice tasting session. The participants were rice consumers and mostly thought they were aware of the health benefits of brown rice. The demographics are shown in Table 2.

### 3.2. Liking (9-Point Hedonic Scale) of Brown and White Rice Varieties before Tasting

**Figure 1:**
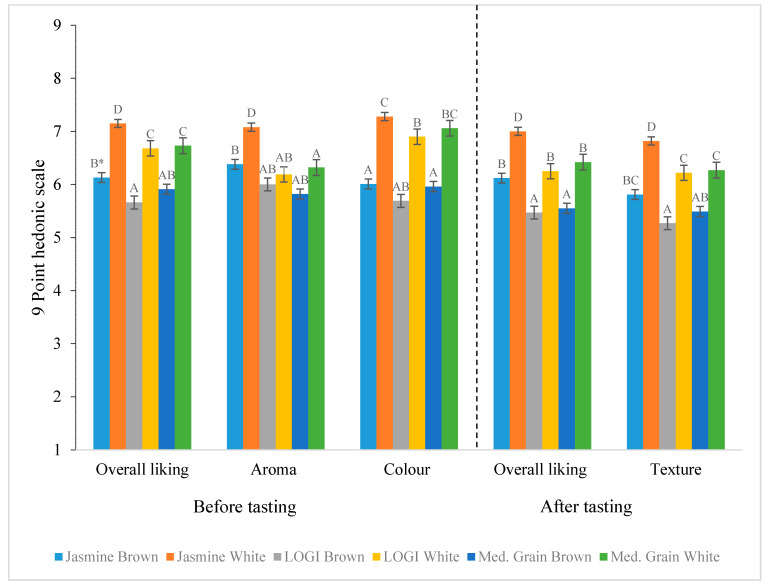
Mean liking (9-point hedonic scale, 1 = extremely disliked to 9 = extremely liked) of sensory attributes for rice varieties. * Different letters, shown as A–D, within attribute are statistically significantly different (*p* < 0.05).

In the result section, rice variety refers to the different varieties which were tested (i.e., Jasmine white, Jasmine brown, Low GI white, Low GI brown, Medium grain white, and Medium grain brown) and rice type refers to brown and white rice. The results (Table 3) indicate that there was a main effect of rice varieties and their types (i.e., brown vs. white) on overall liking before tasting the rice samples (*p* < 0.05). However, there was no statistically significant interaction between rice variety (i.e., Jasmine, Low GI, Medium Grain) and rice type. This means that white rice was preferred over brown rice, regardless of the rice variety (*p* < 0.05) (see Figure 1). Pairwise comparisons show that Jasmine white rice was more liked than any of the other rice varieties (*p* < 0.05), while liking of Low GI white and Medium grain was not statistically significantly different (represented with shared letter “C”). Likewise, no difference was observed between the overall liking of brown rice varieties.

For aroma liking, there was a significant difference (Table 3) between rice varieties and their types (i.e., brown vs. white) before tasting the rice samples. The differences in mean values of Low GI and Medium Grain were −0.6, 95% CI (−0.8, −0.5) and −0.7, 95% CI (−0.9, −0.5), respectively, when compared with Jasmine rice (a reference sample). The interaction between rice variety (i.e., Jasmine, Low GI, Medium Grain) and rice type was also statistically significant, meaning that the aroma of white rice was preferred over brown rice, regardless of the rice variety (*p* < 0.05) (see Figure 1). Pairwise comparisons show that the aroma of Jasmine white rice was more liked than any of the other rice varieties (i.e., Jasmine white rice has the highest mean 7.0, 95% CI (6.9, 7.3) and Medium Grain brown has lowest mean 5.8, 95% CI (5.5, 6.1)). On the other hand, liking of Low GI white and Medium grain was not statistically significantly different (represented with shared letter “AB”). Similarly, no difference was observed between the aroma liking of Low GI and Medium Grain brown rice varieties.

The rice varieties and their types (i.e., brown vs. white) were significantly associated with colour liking before tasting the rice samples. The differences in mean values of Low GI and Medium Grain for colour liking were −0.4, 95% CI (−0.5, −0.2) and −0.1, 95% CI (−0.3, −0.04) respectively when compared with Jasmine rice (a reference sample). However, the interaction between rice variety (i.e., Jasmine, Low GI, Medium Grain) and rice type was not statistically significant. That is, the colour of white rice was liked more than the colour of brown rice, regardless of the rice variety (see Figure 1). Pairwise comparisons show that there was no difference in colour liking of Jasmine white and Medium Grain white rice (represented with shared letter “C”). Likewise, no difference was observed in colour liking of Jasmine brown, Low GI brown and Medium Grain brown (represented with shared letter “A”).

#### Liking (9-Point Hedonic Scale) of Brown and White Rice Varieties after Tasting

The results show rice variety and rice type (i.e., brown and white) after tasting significantly affect liking (see Table 4). Jasmine rice was liked more than Low GI and Medium Grain rice. For all rice varieties, white rice was preferred over brown rice (mean difference = 0.8, 95% CI (0.6, 1.1). The significant interaction between rice varieties and rice types (i.e., brown and white) shows that Jasmine white rice was liked more than any of the other brown and white rice varieties (see Figure 1). The pairwise comparisons show that no difference was observed between Low GI white rice, Medium Grain white rice and Jasmine brown rice in overall liking after tasting.

There was a significant correlation between rice variety and rice type on texture liking (*p* < 0.05) after tasting rice samples (Table 4). This means that the texture of Jasmine rice was liked more than the texture of Low GI and Medium Grain. The mean liking of Low GI and Medium Grain rice were reduced by −0.6, 95% CI (−0.8, −0.4) and −0.4, 95% CI (−0.7, −0.2), respectively, when compared with Jasmine rice (a reference sample). Likewise, the texture of white rice was preferred over brown rice, regardless of rice varieties (mean difference = 0.91, 95% CI (0.6, 1.2). The significant interaction between rice varieties and rice types also indicates that the texture of Jasmine white rice was liked more than the texture of any of the other brown and white rice varieties (see Figure 1). The pairwise comparisons show that no difference was observed between Low GI white rice, Medium Grain white rice, and Jasmine brown rice in texture liking after tasting. However, the texture liking of brown rice varieties was not statistically different.

### 3.3. Just About Right Attributes and Penalty Analysis

#### 3.3.1. Penalty Analysis of Jasmine Brown and White Rice before Tasting

**Figure 2:**
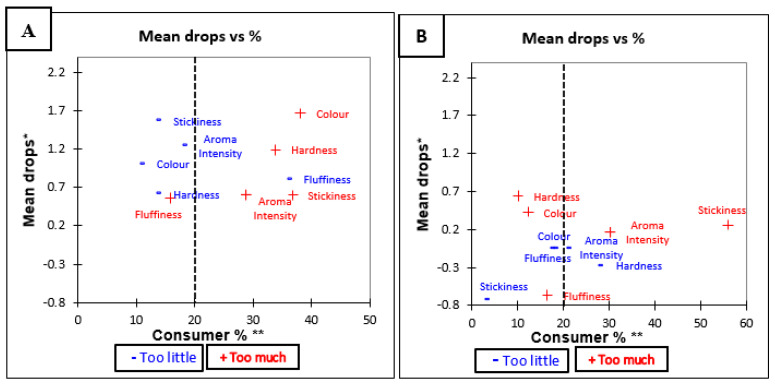
Mean drop plots for Jasmine rice variety before tasting (**A**) Jasmine Brown, and (**B**) Jasmine White. * Mean drop is the decrease in liking compared to the mean liking of those who rated the attribute as JAR. ** Consumer % are the consumers which judged an attribute as not ideal (Just About Right). The attributes with large percentages of consumers and penalties are in top right quadrant of the plot, which illustrates the critical points of the product [34].

Penalty analysis shown in Table 5 indicates that the overall penalty is significant (*p* < 0.05) for all attributes of Jasmine brown rice. This means that the rice was not perceived at optimum level for all attributes tested. The Jasmine brown rice was rated as being too low in aroma, too dark in colour, too hard in texture, too low in fluffiness, and/or too low in stickiness. For Jasmine white rice, the overall penalty (Table 5) was not significant for any of the attributes. This means that across all tested attributes, a deviation from JAR did not have a significant influence on overall liking. The mean drop plot against consumers for each attribute of Jasmine brown and white rice is shown in Figure 2A,B, which visually represents the results of the penalty analysis.

#### 3.3.2. Penalty Analysis of Low GI Brown and White Rice before Tasting

**Figure 3:**
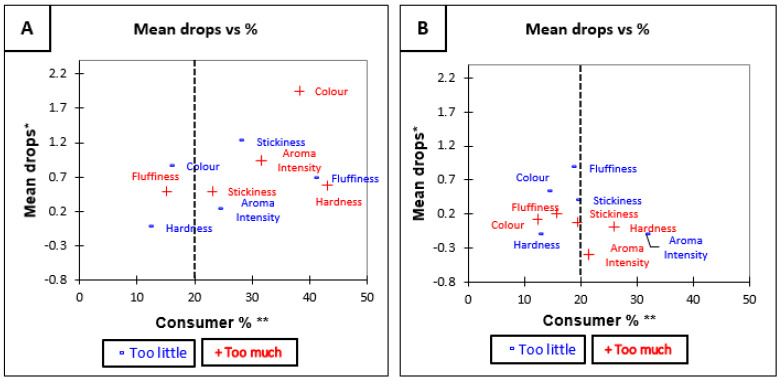
Mean drop plots for Low GI rice variety before tasting (**A**) Low GI Brown and (**B**) Low GI White. * Mean drop is the decrease in liking compared to the mean liking of those who rated the attribute as JAR. ** Consumer % are the consumers which judged an attribute as not ideal (Just About Right). The attributes with large percentages of consumers and penalties are in top right quadrant of the plot, which illustrates the critical points of the product [34].

The overall penalty analysis for Low GI brown rice was significant (*p* < 0.05) for all attributes except “hardness” (*p* = 0.18) (see Table 6). This means that the hardness of Low GI brown rice was the only attribute which was rated as being optimal. The penalty analysis (Table 6) showed that the overall penalty for Low GI white rice was significant for fluffiness (*p* < 0.05). This means that the rating of liking was significantly negatively influenced when participant rated Low GI white as low in fluffiness. Specific changes in liking due to suboptimal attributes are shown in Figure 3A,B which visually represents the penalty analysis of Low GI rice.

#### 3.3.3. Penalty Analysis of Medium Grain Brown and White Rice before Tasting

**Figure 4:**
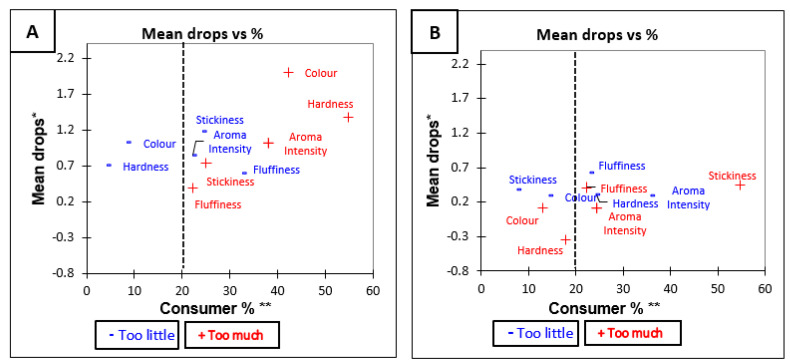
Mean drop plots for Medium Grain rice variety before tasting (**A**) Medium Grain Brown and (**B**) Medium Grain White. * Mean drop is the decrease in liking compared to the mean liking of those who rated the attribute as JAR. ** Consumer % are the consumers which judged an attribute as not ideal (Just About Right). The attributes with large percentages of consumers and penalties are in top right quadrant of the plot, which illustrates the critical points of the product [34].

The results of the penalty analysis (Table 7) for Medium Grain brown rice show that the overall liking was significantly (*p* < 0.05) influenced when the majority of the participants considered that aroma, colour, and hardness were not at optimum level, the attributes were too high in aroma, too dark in colour, and too hard in texture. Similarly, the overall penalty (Table 7) for Medium Grain white rice was significant for fluffiness (*p* = 0.02). That means that for fluffiness, the deviations from the Just about right level have a significant impact on overall liking. The impact on liking of each attribute is shown in Figure 4A,B.

#### 3.3.4. Penalty Analysis of Jasmine Brown and Jasmine White Rice after Tasting

**Figure 5:**
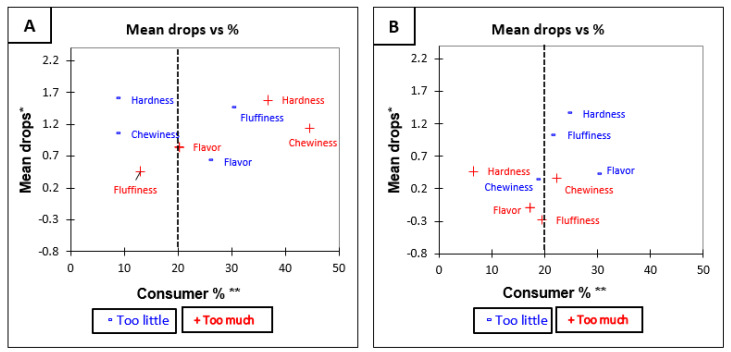
Mean drop plots for Jasmine rice variety after tasting (**A**) Jasmine Brown and (**B**) Jasmine White. * Mean drop is the decrease in liking compared to the mean liking of those who rated the attribute as JAR. ** Consumer % are the consumers which judged an attribute as not ideal (Just About Right). The attributes with large percentages of consumers and penalties are in top right quadrant of the plot, which illustrates the critical points of the product [34].

Penalty analysis (Table 8) indicate that the overall liking was significantly (*p* < 0.05) influenced when participants rated the Jasmine brown rice as not being ideal for flavour, fluffiness, hardness, or chewiness. For Jasmine white rice, the overall penalty (Table 8) was only significant for hardness and not significant for all other attributes after rice tasting. This means that most of the participants considered Jasmine white rice “not hard enough” in texture. The mean drop plot against participants for each attribute by tasting of Jasmine brown and Jasmine white rice is shown in Figure 5A,B.

#### 3.3.5. Penalty Analysis of Low GI Brown and Low GI White Rice after Tasting

**Figure 6:**
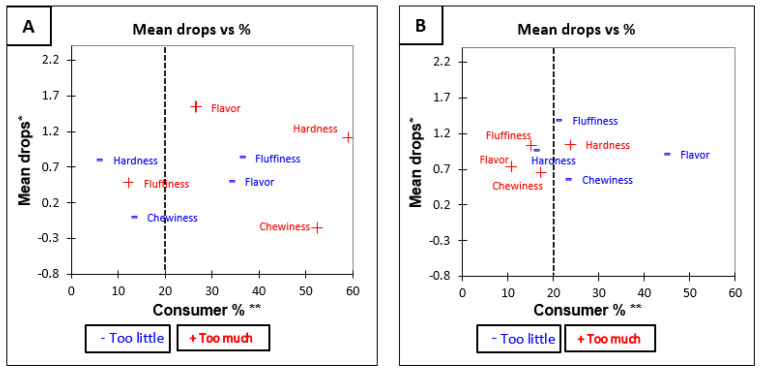
Mean drop plots for Low GI rice variety after tasting (**A**) Low GI Brown and (**B**) Low GI White. * Mean drop is the decrease in liking compared to the mean liking of those who rated the attribute as JAR. ** Consumer % are the consumers which judged an attribute as not ideal (Just About Right). The attributes with large percentages of consumers and penalties are in top right quadrant of the plot, which illustrates the critical points of the product [34].

The penalty analysis (Table 9) of Low GI brown rice by tasting shows that the overall liking was significantly (*p* < 0.05) influenced when most of the participants judged that flavour, hardness, and fluffiness were not optimal in Low GI brown rice. For Low GI white rice, the overall penalty (Table 9) was significant (*p* < 0.05) for all attributes tested. This means that the overall liking was significantly influenced, when the majority of the participants rated Low GI white rice as being not ideal for flavour, fluffiness, hardness, or chewiness. The influence on liking of sensory attributes is shown in Figure 6A,B.

#### 3.3.6. Penalty Analysis of Medium Grain Brown and White Rice after Tasting

**Figure 7:**
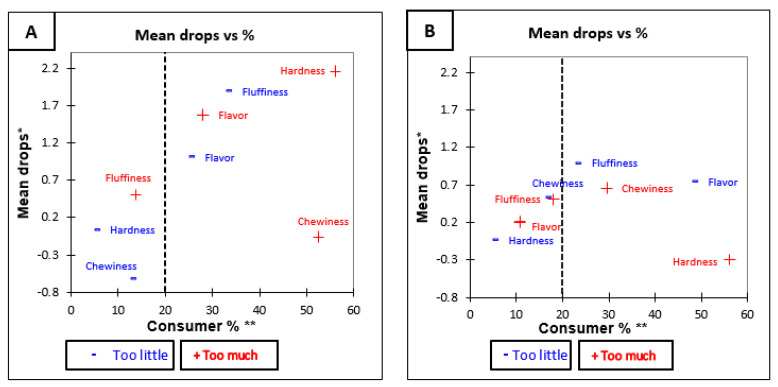
Mean drop plots for Medium Grain rice variety after tasting (**A**) Medium Grain Brown and (**B**) Medium Grain White. * Mean drop is the decrease in liking compared to the mean liking of those who rated the attribute as JAR. ** Consumer % are the consumers which judged an attribute as not ideal (Just About Right). The attributes with large percentages of consumers and penalties are in top right quadrant of the plot, which illustrates the critical points of the product [34].

For Medium Grain brown rice, the penalty analysis (Table 10) showed that the overall liking of Medium Grain brown rice was significantly (*p* < 0.05) influenced when participant rated flavour, fluffiness, and hardness were not at optimum level. Similarly, the overall penalty of Medium Grain white rice was significant for flavour intensity, fluffiness, and chewiness. This means that significant participants perceived Medium Grain white as too low in flavour and fluffiness, and too high in chewiness. The mean drop plots against participants for each attribute of Medium Grain brown rice shown in Figure 7A,B.

## 4. Discussion

This study aimed to identify the consumer liking, sensory attributes, and drivers of liking of brown and white rice varieties. The results suggest that, overall, participants liked Jasmine rice varieties more than Low GI and Medium grain rice varieties. This was also reflected in a higher liking of the aroma, colour, and texture of Jasmine rice, compared to Low GI and Medium grain rice varieties. However, white rice was preferred over brown rice regardless of rice varieties.

The present study suggests, in line with previous studies [19,24,36,37], that texture, colour, and aroma are important drivers of consumer liking for rice. However, these drivers of liking do not seem to equally explain the differences in liking of white and brown rice. Indeed, differences in aroma mainly explain the difference in liking for white rice varieties and the aroma of Jasmine white rice was liked more than any of the other rice varieties. The most liked white rice (Jasmine rice), contains more of the compound 2-acetyle-1-pyrroline [10] which is known to elicit a distinctive popcorn/pandan aroma [3,38,39,40] that has a strong impact on consumer acceptance of rice [41]. On the other hand, the other white rice (non-fragrant) varieties contain less 2AP [42,43,44] that may have an impact on liking of non-fragrant white rice varieties. This is also reflected in the sensory data of the present study that aroma of Jasmine white rice is an important sensory attribute in predicting consumer liking and acceptance of white rice varieties. Therefore, the aroma of Jasmine white rice was preferred over all other white and brown rice varieties. In contrast to aroma being able to explain liking differences for white rice varieties, aroma does not fully explain differences in liking for brown rice.

Differences between brown rice varieties can be explained by texture (hardness and chewiness). This means that brown rice is considered as too hard and chewy in texture, which is driving the difference between brown rice varieties, whereas Jasmine brown rice was preferred over Low GI and Medium grain brown rice. The results are in line with a previous study conducted on ready-to-eat rice in Korea which concluded that the brown rice was scored less in overall acceptability due to being high in hardness, chewiness, and yellowness [18]. Brown rice hardness in texture is associated with dietary fibre that is present in bran layer [45] whereas, in white rice, polishing removes bran and germ during rice processing [46]. This significantly improves texture liking and consumer acceptance of white rice. In contrast to previous studies, which used a combination of descriptive analysis and hedonic scaling [16,18,19,20], the current study investigated consumer acceptance of rice by utilising 9-Point hedonic scales, JAR scales, and penalty analysis. Penalty analysis is a powerful tool to analyse the decreases in acceptability associated with sensory attributes which are perceived by consumers as being not optional [47,48]. This study also compared a range of brown and white rice varieties which enabled to compare brown and white rice, but also identify the drivers of liking between brown rice varieties as well as the drivers of liking within white. In addition, it is interesting to note that rice texture (hardness) is more important for the consumer acceptance and overall liking of Australian brown rice varieties. This study suggests that the decrease in hardness and chewiness will increase the overall liking of Australian brown rice varieties, which can eventually increase brown rice acceptance and consumption.

Brown rice texture (hardness and chewiness) and colour are the sensory attributes that are driving the difference between white and brown rice varieties. Thus, the texture of brown rice is less liked as compare to white rice regardless of rice varieties, because the majority of participants rated brown rice varieties as too hard and too chewy. However, differences in texture seem to be more important when comparing liking between white and brown rice. This is in line with a study conducted on consumer acceptance of parboiled brown and white rice which reported that white rice was preferred to brown rice because of texture and colour [24]. The results are also in agreement with the study that reported consumer acceptance of white rice varieties in Thailand, in which the participants preferred cooked white rice because of the soft texture [36]. Suwansri and Meullenet (2004) reported that Asian consumers preferred rice with white appearance (colour) and less sticky texture [49]. Similarly, the consumers from South Asia and Middle East did not prefer the brown rice texture [50]. In the present study, the sensory results also suggest that brown rice texture (hardness and chewiness) is the most important sensory attribute that is driving the liking and consumer acceptance of brown rice.

Although this was the first study which investigated consumer acceptance of Australian brown and white rice varieties, there are some limitations which need to be taken into consideration. The participants were mainly living in urban areas and were well educated, with 79% of participants holding undergraduate degree or higher. That may have affected their liking because of their awareness of the brown and white rice varieties which may cause bias in evaluation of rice attributes. For future investigation, the sample (participants) could be recruited from different geographical areas to predict the preference of Australian brown and white rice varieties. It is suggested to conduct future studies with a greater focus on the texture attributes of brown rice. To identify the variability in the texture of brown rice, different cooking methods and water to rice ratios are recommended. In addition, the instrumental analysis (colour and texture analyser) can be considered for the better understanding of texture attributes of brown and white rice varieties.

## 5. Conclusions

Texture is the most important sensory attribute which explains the difference in liking between brown and white rice, whereas differences in aroma best explain the variation in liking of white rice. Therefore, to increase the acceptance and consumption of brown rice, development needs to mainly focus on the improvement of the texture acceptance of brown rice. Future research is needed to investigate if an increased water absorption, milling process, packaging, and storage of brown rice can positively improve the texture and subsequently increase consumer acceptance.

## Figures and Tables

**Table 1:** Selected Australian rice varieties.

Rice Varieties	Samples (Types)	Water to Rice Ratio
Jasmine (Kyeema)	Brown rice	2:1
Jasmine (Kyeema)	White rice	1.5:1
Low GI (Doongara)	Brown rice	2:1
Low GI (Doongara)	White rice	1.5:1
Medium grain (Amaroo)	Brown rice	2:1
Medium grain (Amaroo)	White rice	1.5:1

**Table 2:** Demographics of the participants for the rice tasting study.

	Demographics	Participants
Age groups	Age 18–30 years	56%
	Age 31–45 years	28%
	Age 46 years and above	16%
Rice consumers	Brown rice	10%
	White rice	37%
	Brown and white rice	53%
Brown rice health benefits_perceived knowledge	Aware enough	67%
	Not aware enough	33%
Education	High school certificate/Diploma	21%
	Bachelor and above	79%

**Table 3:** Linear mix model (repeated measures) ANOVA table for brown and white rice varieties before tasting.

Overall Liking			Aroma Liking		Colour Liking	
	Mean (95% CI)	Mean (95% CI)	Chi-Square (df) *p* Value	Mean (95% CI)	Chi-Square (df) *p* Value	
Rice variety						
Jasmine (ref)	6.6 (6.4–6.8)	26.50 (2) *p* < 0.0001	6.7 (6.5–6.9)	53.84 (2) *p* ≤ 0.0001	6.6 (6.4, 6.8)	20.08 (2) *p* ≤ 0.0001
Low GI	6.2 (6.0–6.4) ^1^		6.1 (5.9–6.3) ^1^		6.2 (6.1, 6.5) ^1^	
Medium Grain	6.3 (6.1–6.5) ^1^		6.1 (5.9–6.3) ^1^		6.5 (6.3, 6.7)	
Rice type						
Brown (ref)	5.9 (5.6–6.2)	45.50 (1) *p* < 0.0001	6.1 (5.8–6.3)	13.93 (1) *p* ≤ 0.0002	5.9 (5.6–6.2)	50.85 (1) *p* ≤ 0.0001
White	6.9 (6.7–7.0) ^1^		6.5 (6.3–6.7) ^1^		7.0 (6.9–7.3) ^1^	
Pairwise comparisons of variety and rice type (Bonferroni groups) ^2^						
Jasmine White	7.2 (6.9, 7.4)D	1.69 (2) *p* = 0.43	7.0 (6.9, 7.3)D	6.94 (2) *p* = 0.03	7.3 (7.1, 7.5)C	1.21 (2) *p* = 0.55
Low GI White	6.7 (6.5, 6.9)C		6.2 (5.9, 6.4)AB		6.9 (6.7, 7.1)B	
Medium Grain White	6.7 (6.5, 7.0)C		6.3 (6.1, 6.6)AB		7.0 (6.9, 7.2)BC	
Jasmine Brown	6.1 (5.8, 6.4)B		6.4 (6.1, 6.7)B		6.0 (5.7, 6.3)A	
Low GI Brown	5.7 (5.3, 6.0)A		6.0 (5.7, 6.3)AB		6.0 (5.7, 6.3)AB	
Medium Grain Brown	5.9 (5.6, 6.2)AB		5.8 (5.5, 6.1)A		5.8 (5.5, 6.1)A	

^1^ Statistically significant (*p* < 0.001) from the reference (ref). ^2^ rice variety with different letters are statistically significant different (*p* < 0.05).

**Table 4:** Mix model (repeated measures) ANOVA table for brown and white rice varieties after tasting.

Overall Liking		Texture Liking	
	Mean (95% CI)	Mean (95% CI)	Chi-Square (df) *p* Value
Rice variety			
Jasmine (ref)	6.5 (6.3, 6.8)	6.3 (6.1, 6.6)	25.67(2) *p ≤* 0.0001
Low GI	5.9 (5.6, 6.1) ^1^	5.7 (5.5, 6.1) ^1^	
Medium Grain	6.0 (5.8, 6.2) ^1^	5.9 (5.6, 6.1) ^1^	
Rice type			
Brown (ref)	5.7 (5.4, 6.0)	5.5 (5.2, 5.8)	35.25(1) *p ≤* 0.0001
White	6.6 (6.4, 6.8) ^1^	6.4 (6.2, 6.6) ^1^	
Pairwise comparisons of variety and rice type (Bonferroni groups) ^2^			
Jasmine White	7.0 (6.7, 7.3)D	6.8 (6.5, 7.1)D	1.11(2) *p* = 0.57
Low GI White	6.3 (6.0, 6.5)B	6.2 (5.9, 6.5)C	
Medium Grain White	6.4 (6.2, 6.7)B	6.3 (6.0, 6.6)C	
Jasmin Brown	6.1 (5.8, 6.4)B	5.8 (5.5, 6.1)BC	
Low GI Brown	5.5 (5.2, 5.8)A	5.3 (5.0, 5.6)A	
Medium Grain Brown	5.5 (5.2, 5.9)A	5.5 (5.1, 5.8)AB	

^1^ statistically significant (*p* < 0.001) from the reference (ref). ^2^ rice variety with different letters are statistically significant different (*p* < 0.05).

**Table 5:** The Penalty analysis and JAR variables (before tasting) for Jasmine brown and white rice.

Rice	Attribute	Sensory Test	Correlation Coefficient ^a^	Level	Selection% ^b^	Mean ^c^	Mean Drop ^d^	Penalty ^e^
**Jasmine Brown**	Aroma	Smell	0.12	Too low	17.99	5.28	1.25	
				JAR	53.24	6.53		0.85 **
				Too high	28.78	5.93	0.60	
	Colour	Visual	−0.35	Too light	10.79	5.87	1.0	
				JAR	51.08	6.87		1.52 *
				Too dark	38.13	5.21	1.67	
	Hardness	Handling	−0.22	Not hard enough	13.67	6.0	0.62	
				JAR	52.52	6.62		1.03 *
				Too hard	33.81	5.43	1.19	
	Fluffiness	Handling	0.16	Too low	35.97	5.70	0.81	
				JAR	48.20	6.50		0.73 **
				Too much	15.83	5.96	0.55	
	Stickiness	Handling	0.01	Too low	13.67	5.0	1.57	
				JAR	49.64	6.57		0.87 **
				Too much	36.69	5.96	0.60	
**Jasmine White**	Aroma	Smell	0.02	Too low	20.86	7.24	−0.05	
				JAR	48.92	7.19		0.08
				Too high	30.22	7.02	0.17	
	Colour	Visual	−0.09	Too light	17.27	7.25	−0.06	
				JAR	70.50	7.19		0.15
				Too dark	12.23	6.77	0.43	
	Hardness	Handling	−0.12	Not hard enough	28.06	7.41	−0.27	
				JAR	61.87	7.14		−0.30
				Too hard	10.07	6.50	0.64	
	Fluffiness	Handling	0.13	Too low	17.99	7.08	−0.05	
				JAR	65.47	7.03		−0.34
				Too much	16.55	7.70	−0.66	
	Stickiness	Handling	−0.04	Too low	2.88	8.0	−0.72	
				JAR	41.01	7.28		0.22
				Too much	56.12	7.01	0.27	

^a^ The impact of JAR variables for Jasmine brown and white rice on the overall liking (Spearman’s correlation coefficient with a significance level α = 0.05). The correlation coefficients (between JAR attributes and overall liking) show how much JAR attributes have impacted (“low” or “high”) on overall liking for rice samples [34]. When the correlation is positive, the “too little” has a bigger impact than the “too much”, and vice versa for the negative correlations. If correlation is “0” for a JAR attribute, then that attribute would have a strong impact on overall liking [35]. ^b^ Selection % is the percentage of consumers who rate the rice as too low, JAR, or too high on a given attribute. ^c^ Mean is the mean overall liking (9-point hedonic scale) of consumers who rated a given attribute as too low, JAR, or too high. ^d^ Mean drop is the decrease in liking compared to the mean liking of those who rated the attribute as JAR. ^e^ Penalty is a weighted difference between means (mean liking of JAR category minus the mean of liking for other two levels (too low and too high) taken together). * *p* ≤ 0.001, ** *p* ≤ 0.05.

**Table 6:** The Penalty analysis and JAR variables (before tasting) for Low GI brown and white rice.

Rice	Variable	Sensory Test	Correlation Coefficient ^a^	Level	Selection% ^b^	Mean ^c^	Mean Drop ^d^	Penalty ^e^
**Low GI Brown**	Aroma	Smell	−0.15	Too low	24.46	5.79	0.22	
				JAR	43.88	6.02		0.63 **
				Too high	31.65	5.07	0.95	
	Colour	Visual	−0.38	Too light	15.83	5.68	0.87	
				JAR	46.04	6.55		1.64 *
				Too dark	38.13	4.59	1.96	
	Hardness	Handling	−0.18	Not hard enough	12.23	5.94	−0.04	
				JAR	44.60	5.90		0.44
				Too hard	43.17	5.33	0.57	
	Fluffiness	Handling	0.14	Too low	41.01	5.33	0.68	
				JAR	43.88	6.01		0.63 **
				Too much	15.11	5.52	0.49	
	Stickiness	Handling	0.16	Too low	28.06	4.90	1.22	
				JAR	48.92	6.12		0.89 **
				Too much	23.02	5.63	0.49	
**Low GI White**	Aroma	Smell	0.06	Too low	31.65	6.66	−0.09	
				JAR	46.76	6.57		−0.22
				Too high	21.58	6.97	−0.40	
	Colour	Visual	0.08	Too light	14.39	6.25	0.53	
				JAR	73.38	6.78		0.34
				Too dark	12.23	6.65	0.13	
	Hardness	Handling	0.02	Not hard enough	12.95	6.78	−0.11	
				JAR	61.15	6.67		−0.03
				Too hard	25.90	6.67	0.004	
	Fluffiness	Handling	0.17	Too low	18.71	6.0	0.88	
				JAR	65.47	6.88		0.57 **
				Too much	15.83	6.68	0.20	
	Stickiness	Handling	0.06	Too low	19.42	6.37	0.41	
				JAR	61.15	6.78		0.24
				Too much	19.42	6.70	0.07	

^a^ The impact of JAR variables for Jasmine brown and white rice on the overall liking (Spearman’s correlation coefficient with a significance level α = 0.05). The correlation coefficients (between JAR attributes and overall liking) show how much JAR attributes have impacted (“low” or “high”) on overall liking for rice samples. When the correlation is positive, the “too little” has a bigger impact than the “too much”, and vice versa for the negative correlations. If correlation is “0” for a JAR attribute, then that attribute would have a strong impact on overall liking [35]. ^b^ Selection % is the percentage of consumers who rate the rice as too low, JAR, or too high on a given attribute. ^c^ Mean is the mean overall liking (9-point hedonic scale) of consumers who rated a given attribute as too low, JAR, or too high. ^d^ Mean drop is the decrease in liking compared to the mean liking of those who rated the attribute as JAR. ^e^ Penalty is a weighted difference between means (mean liking of JAR category minus the mean of liking for other two levels (too low and too high) taken together). * *p* ≤ 0.001, ** *p* ≤ 0.05.

**Table 7:** The Penalty analysis and JAR variables (before tasting) for Medium grain brown and Medium grain white rice.

Rice	Variable	Sensory Test	Correlation Coefficient ^a^	Level	Selection% ^b^	Mean ^c^	Mean Drop ^d^	Penalty ^e^
**Medium Grain Brown**	Aroma	Smell	−0.11	Too low	22.30	5.65	0.85	
				JAR	39.57	6.49		0.96 **
				Too high	38.13	5.47	1.02	
	Colour	Visual	−0.44	Too light	8.63	5.83	1.02	
				JAR	48.92	6.85		1.84 *
				Too dark	42.45	4.85	2.01	
	Hardness	Handling	−0.39	Not hard enough	4.32	6.0	0.70	
				JAR	41.01	6.70		1.34 *
				Too hard	54.68	5.32	1.39	
	Fluffiness	Handling	0.12	Too low	33.09	5.61	0.59	
				JAR	44.60	6.19		0.51
				Too much	22.30	5.81	0.39	
	Stickiness	Handling	0.07	Too low	24.46	5.21	1.18	
				JAR	50.36	6.39		0.95 *
				Too much	25.18	5.66	0.73	
**Medium Grain White**	Aroma	Smell	0.04	Too low	35.97	6.58	0.28	
				JAR	39.57	6.86		0.21
				Too high	24.46	6.74	0.12	
	Colour	Visual	0.05	Too light	14.39	6.50	0.28	
				JAR	72.66	6.78		0.20
				Too dark	12.95	6.67	0.12	
	Hardness	Handling	0.14	Not hard enough	24.46	6.44	0.30	
				JAR	57.55	6.74		0.26
				Too hard	17.99	7.08	−0.34	
	Fluffiness	Handling	0.08	Too low	23.02	6.34	0.62	
				JAR	54.68	6.96		0.52 **
				Too much	22.30	6.55	0.41	
	Stickiness	Handling	−0.13	Too low	7.91	6.64	0.36	
				JAR	37.41	7.0		0.44
				Too much	54.68	6.55	0.45	

^a^ The impact of JAR variables for Jasmine brown and white rice on the overall liking (Spearman’s correlation coefficient with a significance level α = 0.05). The correlation coefficients (between JAR attributes and overall liking) show how much JAR attributes have impacted (“low” or “high”) on overall liking for rice samples. When the correlation is positive, the “too little” has a bigger impact than the “too much”, and vice-versa for the negative correlations. If correlation is “0” for a JAR attribute, then that attribute would have a strong impact on overall liking [35]. ^b^ Selection % is the percentage of consumers who rate the rice as too low, JAR, or too high on a given attribute. ^c^ Mean is the mean overall liking (9-point hedonic scale) of consumers who rated a given attribute as too low, JAR, or too high. ^d^ Mean drop is the decrease in liking compared to the mean liking of those who rated the attribute as JAR. ^e^ Penalty is a weighted difference between means (mean liking of JAR category minus the mean of liking for other two levels (too low and too high) taken together). * *p* ≤ 0.001, ** *p* ≤ 0.05.

**Table 8:** The Penalty analysis and JAR variables (after tasting) for Jasmine brown and Jasmine white rice.

Rice	Variable	Correlation Coefficient ^a^	Level	Selection% ^b^	Mean ^c^	Mean Drop ^d^	Penalty ^e^
**Jasmine Brown**	Flavour	0.02	Too low	25.90	5.83	0.62	
			JAR	53.96	6.45		0.72 **
			Too high	20.14	5.61	0.85	
	Fluffiness	0.27	Too low	30.22	5.17	1.45	
			JAR	56.83	6.62		1.15 *
			Too much	12.95	6.17	0.45	
	Hardness	−0.26	Not hard enough	8.63	5.25	1.59	
			JAR	54.68	6.84		1.59 *
			Too hard	36.69	5.26	1.59	
	Chewiness	−0.20	Too low	8.63	5.67	1.06	
			JAR	46.76	6.73		1.13 *
			Too much	44.60	5.58	1.14	
**Jasmine White**	Flavour	0.20	Too low	30.22	6.69	0.42	
			JAR	52.52	7.11		0.23
			Too high	17.27	7.21	−0.10	
	Fluffiness	0.25	Too low	21.58	6.13	1.04	
			JAR	58.99	7.17		0.42
			Too much	19.42	7.44	−0.27	
	Hardness	0.27	Not hard enough	24.46	6.00	1.37	
			JAR	69.06	7.37		1.18 *
			Too hard	6.47	6.89	0.48	
	Chewiness	−0.03	Too low	18.71	6.81	0.34	
			JAR	58.99	7.15		0.36
			Too much	22.30	6.77	0.37	

^a^ The impact of JAR variables for Jasmine brown and white rice on the overall liking (Spearman’s correlation coefficient with a significance level α = 0.05). The correlation coefficients (between JAR attributes and overall liking) show how much JAR attributes have impacted (“low” or “high”) on overall liking for rice samples. When the correlation is positive, the “too little” has a bigger impact than the “too much”, and vice-versa for the negative correlations. If correlation is “0” for a JAR attribute, then that attribute would have a strong impact on overall liking [35]. ^b^ Selection % is the percentage of consumers who rate the rice as too low, JAR, or too high on a given attribute. ^c^ Mean is the mean overall liking (9-point hedonic scale) of consumers who rated a given attribute as too low, JAR, or too high. ^d^ Mean drop is the decrease in liking compared to the mean liking of those who rated the attribute as JAR. ^e^ Penalty is a weighted difference between means (mean liking of JAR category minus the mean of liking for other two levels (too low and too high) taken together). * *p* ≤ 0.001, ** *p* ≤ 0.05.

**Table 9:** The Penalty analysis and JAR variables (after tasting) for Low GI brown and Low GI white rice.

Rice	Variable	Correlation Coefficient ^a^	Level	Selection% ^b^	Mean ^c^	Mean Drop ^d^	Penalty ^e^
**Low GI Brown**	Flavour	−0.16	Too low	33.81	5.55	0.50	
			JAR	39.57	6.06		0.96 **
			Too high	26.62	4.51	1.54	
	Fluffiness	0.19	Too low	35.97	5.0	0.83	
			JAR	51.80	5.83		0.74 **
			Too much	12.23	5.35	0.48	
	Hardness	−0.33	Not hard enough	5.76	5.38	0.81	
			JAR	35.25	6.18		1.10 *
			Too hard	58.99	5.06	1.12	
	Chewiness	0.02	Too low	12.95	5.39	0.01	
			JAR	34.53	5.40		−0.12
			Too much	52.52	5.55	−0.15	
**Low GI White**	Flavour	0.21	Too low	44.60	5.82	0.92	
			JAR	44.60	6.74		0.89 *
			Too high	10.79	6.0	0.74	
	Fluffiness	0.12	Too low	20.86	5.31	1.39	
			JAR	64.03	6.70		1.24 *
			Too much	15.11	5.67	1.03	
	Hardness	−0.11	Not hard enough	15.83	5.68	0.97	
			JAR	60.43	6.66		1.02 *
			Too hard	23.74	5.61	1.05	
	Chewiness	0.04	Too low	23.02	5.94	0.56	
			JAR	59.71	6.49		0.60 **
			Too much	17.27	5.83	0.66	

^a^ The impact of JAR variables for Jasmine brown and white rice on the overall liking (Spearman’s correlation coefficient with a significance level α = 0.05). The correlation coefficients (between JAR attributes and overall liking) show how much JAR attributes have impacted (“low” or “high”) on overall liking for rice samples. When the correlation is positive, the “too little” has a bigger impact than the “too much”, and vice versa for the negative correlations. If correlation is “0” for a JAR attribute, then that attribute would have a strong impact on overall liking [35]. ^b^ Selection % is the percentage of consumers who rate the rice as too low, JAR, or too high on a given attribute. ^c^ Mean is the mean overall liking (9-point hedonic scale) of consumers who rated a given attribute as too low, JAR, or too high. ^d^ Mean drop is the decrease in liking compared to the mean liking of those who rated the attribute as JAR. ^e^ Penalty is a weighted difference between means (mean liking of JAR category minus the mean of liking for other two levels (too low and too high) taken together). * *p* ≤ 0.001, ** *p* ≤ 0.05.

**Table 10:** The Penalty analysis and JAR variables (after tasting) for Medium Grain brown and white rice.

Rice	Variable	Correlation Coefficient ^a^	Level	Selection% ^b^	Mean ^c^	Mean Drop ^d^	Penalty ^e^
**Medium Grain Brown**	Flavour	−0.11	Too low	25.18	5.23	1.02	
			JAR	46.76	6.25		1.31 *
			Too high	28.06	4.67	1.58	
	Fluffiness	0.34	Too low	33.09	4.35	1.89	
			JAR	53.24	6.24		1.49 *
			Too much	13.67	5.74	0.50	
	Hardness	−0.52	Not hard enough	5.04	6.71	0.05	
			JAR	38.85	6.76		1.98 *
			Too hard	56.12	4.60	2.16	
	Chewiness	−0.07	Too low	12.95	6.06	−0.62	
			JAR	34.53	5.44		−0.17
			Too much	52.52	5.49	−0.05	
**Medium Grain White**	Flavour	0.22	Too low	48.20	6.06	0.75	
			JAR	41.01	6.81		0.65 **
			Too high	10.79	6.60	0.21	
	Fluffiness	0.13	Too low	23.02	5.75	0.99	
			JAR	58.99	6.74		0.78 **
			Too much	17.99	6.24	0.50	
	Hardness	0.11	Not hard enough	5.04	6.29	−0.03	
			JAR	38.85	6.26		−0.27
			Too hard	56.12	6.55	−0.29	
	Chewiness	−0.07	Too low	16.55	6.17	0.53	
			JAR	53.96	6.71		0.61 **
			Too much	29.50	6.05	0.66	

^a^ The impact of JAR variables for Jasmine brown and white rice on the overall liking (Spearman’s correlation coefficient with a significance level α = 0.05). The correlation coefficients (between JAR attributes and overall liking) show how much JAR attributes have impacted (“low” or “high”) on overall liking for rice samples. When the correlation is positive, the “too little” has a bigger impact than the “too much”, and vice versa for the negative correlations. If correlation is “0” for a JAR attribute, then that attribute would have a strong impact on overall liking [35]. ^b^ Selection % is the percentage of consumers who rate the rice as too low, JAR, or too high on a given attribute. ^c^ Mean is the mean overall liking (9-point hedonic scale) of consumers who rated a given attribute as too low, JAR, or too high. ^d^ Mean drop is the decrease in liking compared to the mean liking of those who rated the attribute as JAR. ^e^ Penalty is a weighted difference between means (mean liking of JAR category minus the mean of liking for other two levels (too low and too high) taken together). * *p* ≤ 0.001, ** *p* ≤ 0.05.

## Data Availability

The datasets used and/or analysed during the current study are available from the corresponding author on reasonable request.

## Abbreviations

JAR	Just About Right
IRRI	International Rice Research Institute
CASS	Centre for Analytical Sensory and Safety
ANOVA	Analysis of Variance
XLSTAT	Name of Statistical Sensory Software
CI	Confidence Interval
Ref	Reference
